# Patterned Dried Blood Spot Cards for the Improved Sampling of Whole Blood

**DOI:** 10.1021/acsmeasuresciau.1c00031

**Published:** 2021-09-17

**Authors:** Keith R. Baillargeon, Jessica C. Brooks, Philip R. Miljanic, Charles R. Mace

**Affiliations:** Department of Chemistry and Laboratory for Living Devices, Tufts University, Medford, Massachusetts 02155, United States

**Keywords:** paper microfluidics, paper analytical devices, μPAD, dried blood spot cards, point-of-care, bioanalysis, hematology

## Abstract

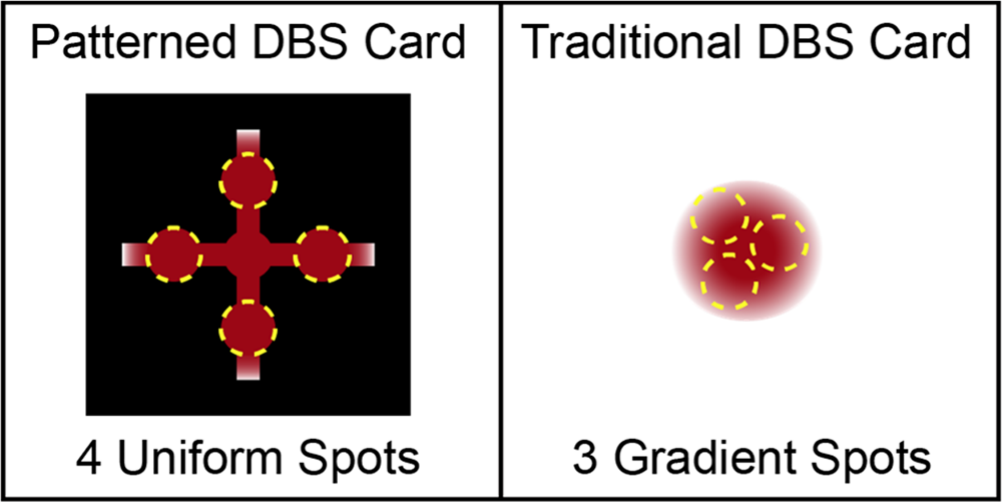

Dried blood spot (DBS) cards perform many functions for sampling blood that is intended for subsequent laboratory analysis, which include: (i) obviating the need for a phlebotomist by using fingersticks, (ii) enhancing the stability of analytes at ambient or elevated environmental conditions, and (iii) simplifying the transportation of samples without a cold chain. However, a significant drawback of standard DBS cards is the potential for sampling bias due to unrestricted filling caused by the hematocrit of blood, which often limits quantitative or reproducible measurements. Alternative microsampling technologies have minimized or eliminated this bias by restricting blood distribution, but these approaches deviate from clinical protocols and present a barrier to broad adoption. Herein, we describe a patterned dried blood spot (pDBS) card that uses wax barriers to control the flow and restrict the distribution of blood to provide enhanced sampling. These patterned cards reproducibly fill four replicate extraction zones independent of the hematocrit effect. We demonstrate a 3-fold improvement in accuracy for the quantitation of hemoglobin using pDBS cards compared to unpatterned cards. Patterned cards also facilitate the near quantitative recovery (ca. 95%) of sodium with no evidence of a statistically significant difference between dried and liquid blood samples. Similarly, the recovery of select amino acids was conserved in comparison to a recent report with improved intercard precision. We anticipate that this approach presents a viable method for preparing and storing samples of blood in limited resource settings while maintaining current clinical protocols for processing and analyzing dried blood spots.

## Introduction

Blood is a complex matrix, comprising cellular and liquid fractions, that contains a wealth of diagnostically relevant biomarkers, which are inclusive of the cells themselves (e.g., neutrophil count), DNA/RNA (e.g., endogenous or from pathogens), and myriad solutes in plasma (e.g., proteins, metabolites, free amino acids). For these reasons, blood is often thought of as the ideal specimen for evaluating the health status of a patient. Obtaining liquid blood samples in centralized facilities or even local clinics is routine practice. In these settings, a trained phlebotomist will collect milliliter volumes of blood by venipuncture, which can either be immediately processed and tested in the laboratory or stored for future analysis within a defined period of time dependent on storage temperature.^1,2^ However, these same practices face unique challenges at the point-of-care or in resource-limited settings. Specifically, the storage and transportation of liquid blood are complicated by unreliable modes of transportation and inadequate access to cold chain storage. These limitations often require liquid samples to be discarded due to substantial degradation or significant changes to critical hematological indices.

In contrast to liquid samples, storing blood in a porous matrix, such as chromatography paper, enhances analyte stability at ambient or elevated temperatures.^3,4^ Dried blood spot (DBS) cards offer simplified sampling using fingersticks and reliable transportation of dried blood by mail, thus circumventing the need for cold chain storage.^5,6^ Traditional DBS cards, such as the Whatman 903 Protein Saver card, are a simple construction of a single sheet of thick cellulose cardstock affixed to an envelope for sample identification and handling.^7^ Circles are printed onto the surface of the paper using a thin layer of toner to provide guidance for sample application. Fingerstick volumes of blood (e.g., 50–100 μL per spot) are applied to the card and allowed to dry for a minimum of 4 h at ambient conditions (and ideally overnight), rendering the card nonbiohazardous, before they are sealed and shipped through the mail for laboratory analysis.^8^ Self-sampling low volumes of blood without the need for cold chain storage could broadly expand access to basic health information by providing direct-to-consumer testing, facilitating critical population screening, and biobanking efforts.^9^

Although traditional DBS cards offer simple operation, require low volumes of blood, and can be collected outside of the clinic, they are severely limited by usability associated with unrestricted sample application zones. This user error can result in nonuniform or smeared blood spots, which will ultimately impact the quality of subsequent laboratory analysis and represents a considerable barrier for the ubiquitous use of traditional DBS cards.^9,10^ Beyond usability, traditional DBS cards do not account for differences in hematocrit values (Hct)—the ratio of packed red blood cells (RBCs) to total blood sample volume. The normal range of hematocrit spans 36–50% and is affected by variables such as race, sex, age, hydration, and overall health status.^11^ Currently, the hematocrit value must be known prior to analysis for the accurate quantitation of analytes using DBS cards. Whether caused by filling imprecision or hematocrit, the uniformity of how cells and liquid plasma are distributed throughout the paper cardstock has a substantial impact on the overall utility of a DBS card.

The hematocrit effect has been extensively reviewed as the main obstacle to overcome for quantitative analysis using traditional DBS cards.^12−18^ Since the hematocrit value represents the ratio of cellular matter to liquid plasma, blood samples with a high hematocrit value (e.g., 55%) will be more viscous than samples with a low hematocrit value (e.g., 30%). Variation in viscosity results in variable sample flow and distribution through the paper, which negatively impacts the reproducibility of sample volumes obtained from a single, fixed punch extraction from DBS (i.e., sub-punch). Uncontrolled saturation or spreading of blood through the DBS paper can also result in a heterogeneous distribution of analytes throughout the area of the resulting DBS (i.e., volcano effect).^19^ Because analytes are typically eluted from DBS via a sub-punch, any variation in sample volume and distribution will manifest in downstream clinical measurements, causing a lack of precision (i.e., intraspot agreement) or accuracy (i.e., agreement with liquid sample).

Many methods for minimizing the hematocrit effect in traditional DBS cards have previously been reported.^20−22^ Two distinct approaches stand out: (i) whole spot analysis^23,24^ and (ii) assay specific calibrants stored within the paper.^25^ Both present viable options for minimizing the hematocrit effect by quantitative removal of the entire blood spot (dependent on the application of accurate sample volume) or the inclusion of an internal standard at a known concentration to estimate extraction efficiency. However, both methods are limited by the number of tests that can be conducted from a single DBS spot. In each format, samples can only be used to perform a single test due to the complete destruction of the entire dried spot or assay-specific internal standard. Alternatively, three-dimensional blood spheroids eliminate chromatographic effects observed in traditional DBS and reduce the volume of blood required per spot by utilizing functionalized hydrophobic paper.^24^ This approach has successfully demonstrated increased stability of enzymes and labile organic compounds at ambient conditions. Recently, DBS technologies that operate independently of the hematocrit by constricting sample volume have also been described. The ADX Test Card by Accel Diagnostics utilizes a microfluidic network and magnetic beads to collect, distribute, and analyze blood.^26^ The HemaSpot HF comprises precut paper wedges contained within a plastic housing, which hold a finite volume of sample.^27^ Similarly, the HemaPEN^28^ and HemaXis DB10^29^ integrate multiple fixed-volume capillary tubes to standardize the volume of blood applied to a porous matrix. While these devices provide enhanced control over the application of sample volume, they do not conform with current clinical collection or automated punching and elution protocols.

In order to improve the utility of DBS cards with an intent for widespread use, current clinical protocols for sample collection and subsequent analysis should be maintained. Therefore, innovation should build upon the major benefits of traditional DBS technology (i.e., single layer of cardstock). An attractive approach for enhanced sampling is controlling the flow of blood samples in the cardstock with hydrophobic wax barriers.^30^ Defining specific areas for (i) sample addition, (ii) distribution, and (iii) storage by wax patterning presents a method for addressing the limitations of current DBS technologies without creating additional clinical barriers. Ideally, blood sampling would be performed via a self-administered fingerstick, simple collection onto a solid matrix, drying, and delivery to a laboratory for testing without significant degradation of the sample at ambient conditions.

Herein, we describe the creation of patterned dried blood spot (pDBS) cards to address the limitations of traditional DBS cards directly related to the hematocrit effect. Patterning traditional DBS cardstock with hydrophobic wax barriers regulates sample application, distribution, and volume control while operating independently of the hematocrit over a broad range of clinical values (20–60%). A user simply needs to apply a volume of blood to the center of the card and the sample will automatically distribute to four replicate punch zones. Providing more spots for analysis while also maintaining reproducible spreading across physiological hematocrit values can (i) increase the number of technical replicates or (ii) increase the number of clinical assays performed from one sample of whole blood without concern for significant punch-to-punch variation.

We first investigated the capacity of pDBS cards for quantitative sampling by estimating the volume of blood contained in a standard 6 mm paper punch and reported minimal variation even when the sample input deviates from the World Health Organization (WHO) recommended volume of 75 μL.^5^ Next, we demonstrated enhanced usability and spot uniformity independent of the hematocrit for samples collected with pDBS cards compared to traditional, unpatterned cardstock. We highlighted a broad class of analytes to showcase this approach including the quantitation of hemoglobin by UV–vis spectrophotometry, sodium by inductively coupled plasma atomic emission spectroscopy (ICP-AES), and specific amino acids by high-performance liquid chromatography (HPLC). pDBS cards permit the enhanced sampling of small volumes of blood that can be generated from a fingerstick and represent a reproducible method capable of performing multiple tests without requiring multiple sample collections or altering established laboratory workflows. We anticipate the quantitative nature of this self-sampling method of blood collection will empower patients by providing critical, accurate diagnostic information at home or in low-income economies without impacting existing clinical procedures.

## Experimental Design: Card Design and Fabrication

**Figure 1:**
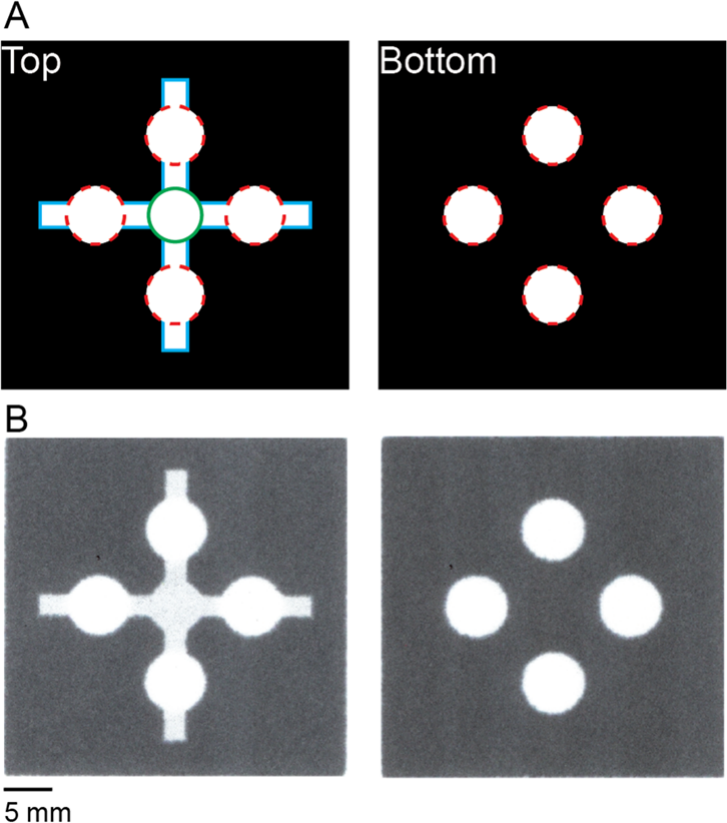
Schematic of a patterned dried blood spot (pDBS) card. (A) Top and bottom of the card are uniquely patterned and include three distinct features: (i) sample addition zone (outlined in green), (ii) lateral distribution channels for sample splitting (outlined in cyan), and (iii) four replicate collection punch zones (outlined in red). Collection punch zones are removed via a standard 6 mm office hole punch prior to analysis. (B) Scanned image of actual pDBS card.

pDBS cards comprise a single layer of cardstock impregnated with wax to form three distinct features: (i) sample addition zone, (ii) lateral distribution channels, and (iii) four replicate, collection punch zones (Figure 1A). We designed our cards to accommodate a sample input volume of 75 μL and output punch diameter of 6 mm in accordance with the WHO recommended specifications for DBS sampling. The design features (e.g., lateral channels) and geometries were informed by our previous experience with whole blood in paper for measuring the hematocrit.^31,32^ Whole blood is transported from the sample addition zone along the lateral channels via capillary action and fills four replicate collection punch zones at the end of each channel. Extending the lateral channels past the collection punch zones allowed for the complete saturation of the punch zone for more accurate sampling compared to traditional DBS cards. Wax printing is typically performed by the direct deposition of wax onto relatively thin (≤250 μm), smooth papers followed by the application of heat to allow the wax to coat the paper fibers.^33^ For papers >250 μm thick, standard printing practices cannot deposit sufficient wax to form complete hydrophobic barriers (Figure S1A). Incomplete barriers resulted in uncontrolled sample flow and represent a challenge for patterning DBS papers. Alternative methods for patterning thick materials with photoresist, paraffin,^34^ and silanes^35^ have been reported previously. However, wax printing offers several advantages such as rapid prototyping, excellent reproducibility, and is suitable for mass production.^33,36^ We utilized a double-sided wax transfer method^37^ to successfully pattern papers commonly used for traditional DBS cards (e.g., Whatman CF-12, Ahlstrom 226, Munktell TFN) (Figure S1B). First, we printed the top and bottom designs onto laminate sheets using a Xerox ColorQube 8580 wax printer. Next, we aligned a sheet of chromatography paper with the top and bottom designs using a custom acrylic alignment jig. Finally, we used a Promo Heat CS-15 T-shirt press (45 s at 137 °C) to transfer the wax from the laminate sheets to the paper to form hydrophobic barriers through the full thickness of the paper.

Patterning each side with a unique design allowed for the partial coating of the cellulose fibers through approximately half the thickness of the paper to reduce the void volume of the sample addition zone and lateral distribution channels in pDBS cards (Figure 1B). This process provided an added benefit of minimizing sample input volume while maximizing sample collection volume from the punch zones. After the addition of whole blood, we dried pDBS cards under ambient conditions in a biosafety cabinet (ca. 16 h), whereby they can be used immediately or sealed in a foil pouch with silica desiccant packets and a humidity indicator card for long-term storage. All data presented herein were collected using pDBS cards fabricated from TFN grade cardstock. We chose to demonstrate the utility of our cards for sampling a range of analytes (e.g., hemoglobin, sodium, and select amino acids) and technique groups (e.g., UV–vis spectrophotometry, ICP-AES, and HPLC).

## Results and Discussion

### Effects of Evaporation on the Quantitation of Hemoglobin

Evaporation at ambient conditions is the driving force for drying samples of blood in DBS cards. Sealing—or partially sealing—sections of our pDBS cards influenced the location and extent of evaporation. Additionally, altering the bottom design of the pDBS card can affect evaporation by controlling the amount of unpatterned card area that is exposed to the environment. We iteratively added or removed a layer of laminate to the top and bottom sides of the pDBS card and evaluated the effects of evaporation on the quantitation of hemoglobin using a modification of the standard Drabkin’s assay (Figure S2). The bottom design either (i) excluded (designs A and B) or (ii) included (designs C and D) the lateral distribution channels. Evaluating these design features across a range of hematocrit is critical for understanding the effects of evaporation since these samples have varying volumes of liquid plasma (e.g., 52.5 μL of plasma in 75 μL of 30% hematocrit blood vs 37.5 μL of plasma in 75 μL of 50% hematocrit blood). Excluding the lateral channels and sample addition zone in the bottom design reduced the total void volume of the unpatterned area and eliminated evaporation from the bottom side of the lateral channels and sample addition zone. Reducing the void volume improved reproducibility for card filling. Further covering the lateral channels on the top side of the pDBS card minimized evaporation along the channel and effectively concentrated the blood sample in the collection punch zones. The preconcentration of blood in the collection punch zones resulted in a higher percent deviation for the quantitation of hemoglobin (Table S1), which we expect is due to the volume dependency of the Drabkin’s assay.^38^ Both designs B and C had comparable performance even though design B included no laminate covering the unpatterned area and design C was completely laminated (except at the sample addition zone). We chose to move forward with design B for two reasons: (i) it yielded the lowest percent error for both 30% and 50% hematocrit samples and (ii) reduced the number of laminate layers necessary, which simplified the manufacturing and operational processes.

### Estimation of Sample Volume in 6 mm Paper Punch

After finalizing the form factor of our pDBS card and minimizing the effect of evaporation through unique bottom patterning, we measured the volume of a dried sample contained in an individual 6 mm paper punch in order to correlate the concentration of an analyte to the total sample of blood. The accurate comparison of liquid reference samples to our pDBS card is dependent on the sample volume contained within a sub-punch. This type of measurement has been accomplished using a variety of methods including ion suppression by liquid chromatography-tandem mass spectrometry^20^ and the electrical conductivity of DBS extract by a ring disk electrode.^10^ We utilized the volume dependency of the Drabkin’s assay to estimate the output sample volume in our pDBS card.^39^ First, we constructed a series of calibration curves (Figure S3A) using liquid hemoglobin standards with varied sample input volumes (3–11 μL) to establish a relationship between the linear slope of the calibration curve and sample volume (Figure S3B). Then, we calibrated our pDBS cards with hemoglobin standards and estimated the sample volume contained in a 6 mm paper sub-punch using the resultant linear relationship and slope of the calibration curve in our pDBS card (Figure S3C). All hemoglobin samples reproducibly filled the pDBS cards (Figure S3D). We estimated that each 6 mm paper sub-punch contained 10.3 ± 0.4 μL of whole blood, representing a total output sample volume of approximately 41.2 μL from an input volume of 75 μL of blood. These data are supported by the calculated theoretical volume (ca. 10.6 μL) occupied in a 6 mm diameter disc of grade TFN (ca. 470 μm thick with a nominal porosity of 80%). The low variation (<5%) observed in the sample volume contained in a paper sub-punch indicated consistent sample distribution in pDBS cards.

Deviating from the recommended sample input volume of 75 μL can negatively impact the quantitation of analytes such as hemoglobin. To simulate under- and overfilling, we applied a range of sample volumes 60–90 μL in 5 μL increments at a single hematocrit (Figure S4A). Our pDBS cards reproducibly filled four replicate punch zones with a sample volume ≥65 μL (Figure S4B). The average deviation for replicate cards with sample input varying ±15 μL was only 12.0% compared to that of the liquid reference sample. This result provided confidence that slight variations in the sample input volume (e.g., from direct addition of a fingerstick rather than sample addition by volumetric pipet) will not substantially impact quantitative results if volumetric sample application is unavailable at the site of collection.

### pDBS Cards Fill Independent of Hematocrit Value

**Figure 2:**
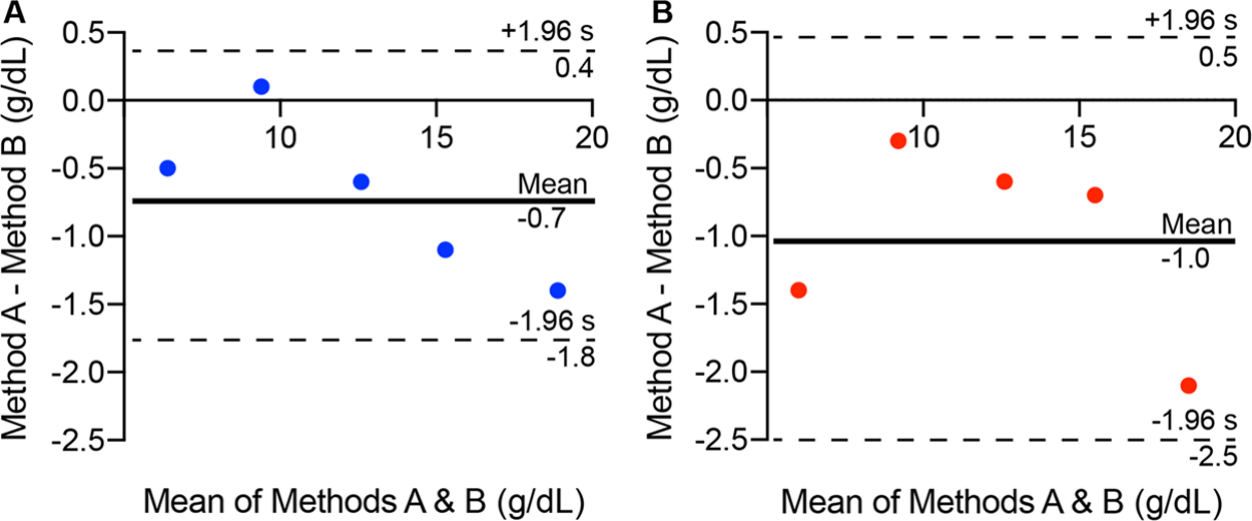
Bland and Altman plots for (A) pDBS card and (B) unpatterned TFN from Table 1. Limits of agreement are represented by dotted lines from −1.96 to +1.96 s.

We aimed to further evaluate the effect of sample input on the quantitation of hemoglobin by surveying the physiological range of hematocrit values (20–60%). We anticipated that controlling the total area of the pDBS card through patterning would minimize the negative effects of variable sample spreading caused by the hematocrit. The direct comparison of pDBS cards and unpatterned TFN clearly demonstrated how the hematocrit influenced the results of standard assays such as the quantitation of hemoglobin (Table 1). Patterned cards yielded ≤7% error across the full range of hematocrit values, while unpatterned cards yielded 3-fold higher percent error at low hematocrit (21% error at 20% hematocrit, Table 1). Inter- and intracard variations (i.e., spot-to-spot variation) were consistent between both card types (Table S2), which suggested that the deviation in the quantitation of hemoglobin can largely be attributed to uncontrolled sample spreading of blood in unpatterned cards. Agreement between pDBS cards (Figure 2A) and unpatterned TFN (Figure 2B) with the reference liquid blood is represented by Bland–Altman plots.^40^ The observed bias was reduced in pDBS cards (−0.7 g/dL) compared to that of TFN (−1.0 g/dL). Similarly, the limit of agreement was narrower for pDBS (2.2 g/dL) than for TFN (3.0 g/dL) in comparison to the reference method.

**Table 1:** Comparison of pDBS Card and Unpatterned Cardstock (TFN) for the Quantitation of Hemoglobin at Various Hematocrit Values^a^

	pDBS card		unpatterned TFN		reference method
Hct (%)	[Hgb] ± SD (g/dL)	error (%)	[Hgb] ± SD (g/dL)	error (%)	[Hgb] ± SD (g/dL)
20	6.2 ± 0.2	–7	5.3 ± 0.1	–21	6.7 ± 0.2
30	9.4 ± 0.2	+1	9.1 ± 0.4	–3	9.4 ± 0.9
40	12.3 ± 0.3	–5	12.3 ± 0.3	–5	12.9 ± 0.5
50	14.7 ± 0.5	–7	15.2 ± 0.5	–4	15.9 ± 0.6
60	18.2 ± 0.5	–7	17.4 ± 0.4	–11	19.6 ± 0.3

a Data represent the average of 20 replicates ± standard deviation. Reference values using reference method (liquid blood) are provided.

**Figure 3:**
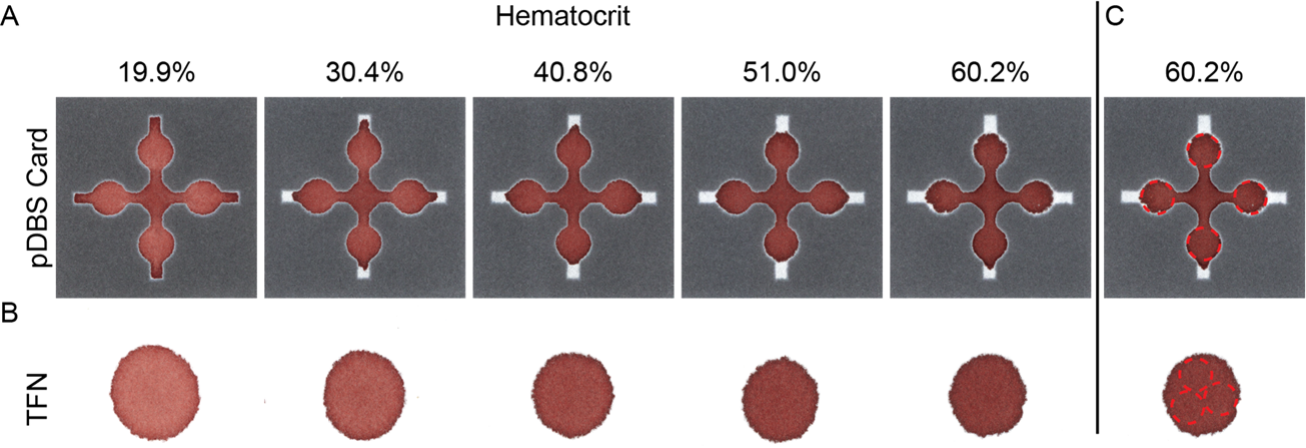
pDBS cards fill uniformly independent of hematocrit value. Representative images of (A) pDBS cards and (B) unpatterned TFN at various hematocrit values (19.9–60.2%, *N* = 5). (C) pDBS cards fill four replicate 6 mm collection punch zones compared to only three replicate punches from unpatterned TFN independent of hematocrit value.

Patterned cards reproducibly filled four replicate collection punch zones (6 mm diameter) across the full range of hematocrit values (Figure 3A). Since pDBS cards filled independently of the hematocrit, four replicate punches can always be collected for analysis and enable more tests to be performed from a single card. In stark contrast, the diameter of the blood spot in unpatterned TFN decreased with increasing hematocrit (20–60% hematocrit) (Figure 3B). A direct consequence of the decreased blood spot diameter in unpatterned DBS is one less technical replicate punch of dried blood under idealized conditions (Figure 3C).

### Quantitation of Sodium by ICP-AES

**Figure 4:**
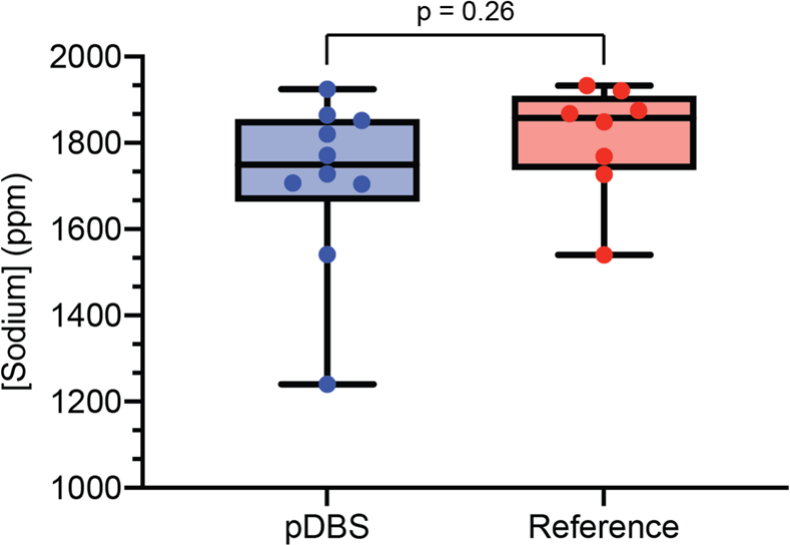
Quantitation of sodium by ICP-AES. Ten replicate samples were prepared using (i) pDBS cards or (ii) reference method with liquid whole blood. A two-tailed Student’s *t* test yielded a *p*-value of 0.26, providing no evidence of a difference in recovered sodium concentration between pDBS card (dried blood) and the reference method (liquid blood).

Blood sodium levels are routinely measured as part of a basic metabolic panel that often includes additional electrolytes such as calcium, chloride, and potassium. The accurate quantitation of sodium is critical for controlling blood pressure and evaluating proper nerve and muscle function.^41^ Additionally, because sodium is found both intra- and extracellularly, it represented an attractive analyte class to further evaluate the quantitative capabilities of pDBS cards. The concentration of sodium in blood samples obtained from pDBS cards (1715 ± 21 ppm) was nearly identical with the concentration in the reference liquid sample (1810 ± 24 ppm), suggesting that there is no apparent loss or evaporative concentration of sodium to the TFN paper (Figure 4). A two-tailed Student’s *t* test yielded a *p*-value of 0.26, providing no evidence of a statistically significant difference in sodium concentration between the dried and liquid blood samples. The clinical reference range for sodium in blood is 135–145 mEq/L.^42^ Both the dried and liquid blood samples fell below the expected range with 74.6 and 78.7 mEq/L sodium, respectively. Both samples were prepared using nitric acid digestion, which included multiple liquid handling and quantitative transfer steps, which could account for the low observed concentrations. While the range of concentrations of sodium extracted from pDBS punches (683.9 ppm) was more dispersed than those from liquid samples of blood (392.4 ppm), the standard deviation was slightly less. The comparison of variances (F-test) yielded a *p*-value of 0.27, indicating no significant difference between the variance of the data sets. Therefore, the precision of pDBS card microsampling could be amenable to use of calibration standards for quantitative results.

### Quantitation of Amino Acids by HPLC

Amino acid analysis via DBS sampling is commonly used for the detection of various inborn errors of amino acid metabolism including phenylketonuria (PKU) in newborns. Efforts to streamline and improve the quantitation of amino acids from DBS have been extensively reported.^43−45^ For demonstrative purposes, we selected three representative hydrophobic amino acids (e.g., tryptophan, leucine, and proline) and one basic—or positively charged—amino acid (e.g., lysine) for analysis. The recovery of each amino acid from pDBS cards was determined by the ratio of extracted analyte concentration (μM) and liquid reference concentration (μM) as analyzed by HPLC. Two distinct sample groups (e.g., 20 and 40% hematocrit) were selected to represent (i) a high liquid-to-cell ratio—which can be prone to underestimating analytes of interest—and (ii) the average hematocrit obtained from our panel of healthy donors, respectively. Each amino acid yielded excellent recovery for both blood sample groups (Table 2). While most samples fell in the range 82–93% recovery, two samples yielded higher concentrations when extracted from pDBS cards compared to liquid reference samples (proline 115%, lysine 102%). Resultant loss and variability in analyte recovery may be attributed to the number of liquid handing steps required to extract, process, and derivatize samples prior to analysis by HPLC. However, all reported values in Table 2 are in agreement with other reports where the recovery of amino acids ranged from 84.2 ± 22.2 to 96.0 ± 12.0%.^46^ Additionally, the evaluation of interassay precision (i.e., card-to-card comparison) demonstrated coefficients of variation (% CV) for tryptophan of 0.8–5.2%, leucine of 2.6–6.7%, proline of 1.0–5.5%, and lysine of 5.4–5.7% (Table S3). These % CV values are considerably improved in comparison to recently reported % CV values for amino acid analysis by traditional DBS sampling using similar methods (e.g., % CV for leucine 8.3–15.3%).^44^ The successful quantitation and improved interassay precision of select amino acids by HPLC supported the enhanced sampling capabilities of pDBS cards.

**Table 2:** Quantitation of Amino Acids by HPLC^a^

	20% hematocrit			40% hematocrit		
	pDBS	reference	recovery (%)	pDBS	reference	recovery (%)
[Trp] ± SEM (μM)	116 ± 6	124 ± 5	93	127 ± 1	137 ± 5	93
[Leu] ± SEM (μM)	30 ± 2	36 ± 2	85	39 ± 1	36 ± 1	90
[Pro] + SEM (μM)	73 ± 4	89 ± 6	82	98 ± 1	85 ± 3	115
[Lys] ± SEM (μM)	37 ± 2	36 ± 2	102	35 ± 2	40 ± 2	87

a Data for liquid blood reference represent the average of five replicates ± standard error of the mean (SEM). Data for pDBS cards represent the average of four replicates ± SEM. Recovery was calculated with respect to liquid blood reference samples.

## Conclusions

We aimed to develop a device that can improve the sampling of whole blood at the point-of-care while maintaining current clinical protocols for DBS analysis. Our approach comprised wax-patterned DBS cardstock to restrict the flow and distribution of whole blood with four defined extraction zones. Controlling the flow of blood in the pDBS card allowed for reproducible filling across the full range of hematocrit values and reduced the sampling bias for pDBS cards compared to unpatterned TFN cardstock. Specifically, the accuracy for the quantitation of hemoglobin with low hematocrit (20%) was improved by 3-fold using pDBS cards. Sampling was further improved by spatially defining extraction zones, which consistently produced four replicate 6 mm diameter sub-punches from a single application of blood (75 μL), independent of the hematocrit value. We designed these cards to accommodate the direct application of fingerstick volumes of blood and modeled ideal conditions by dispensing blood using a volumetric pipet. The highly controlled nature of this method of sample dispensing may be reflective of the conserved inter- and intracard variations reported for both pDBS and traditional DBS cards. We anticipate that the patterned features of pDBS cards will maintain uniform filling and address the reported challenges associated with applying fingersticks to DBS at the point-of-care.

Surveying common DBS analytical techniques such as ICP-AES and HPLC indicated good agreement with liquid reference samples for the quantitation of sodium and select amino acids, respectively. Additionally, we were able to process and analyze samples of whole blood without changing the recommended handling procedures for DBS cards (i.e., amenable to automated punching machines). Standardizing the sample output from pDBS cards could expand the number of tests performed from a single sample collection or permit increased numbers of technical replicates compared to traditional unpatterned DBS cards. Beyond the classes of analytes and techniques demonstrated in this manuscript, quantitative DBS sampling has the potential for myriad applications related to molecular amplification (e.g., screening for viral diseases), nutritional evaluations, immunologic studies, pharmacokinetics, therapeutic drug monitoring, and genetic testing.^47^

Since pDBS cards are exposed to ambient conditions during sample application, spreading, and drying, we expect that performance may vary under certain environmental conditions at the time of collection, as similarly experienced with traditional DBS cards. For example, sample spreading may be reduced due to extremely dry conditions (relative humidity below 10%) or high temperatures, which could cause excessive evaporation. However, this effect is commonplace for DBS technologies and is not identified as a major obstacle for ubiquitous use.^6^ Additionally, hydrophobic wax barriers used in this manuscript are incompatible with organic solvents (e.g., methanol, acetonitrile, ethanol) and harsh surfactants (e.g., Triton X-100 and sodium dodecyl sulfate). Removing paper zones from the pDBS card via punches prior to elution of analyte will avoid leeching wax, or other material used to generate hydrophobic barriers, into the final tested sample. While the pDBS card presented here was used for sampling whole blood, we anticipate that we could expand on this approach to collect and store additional sample types such as saliva, tears, or blood plasma to provide enhanced sampling and quantitative analysis in a workflow that connects the point-of-care to a clinical laboratory infrastructure.

## Supporting Information Available

Discussions of materials and methods used, figures of scanned images of card patterning, quantitation of hemoglobin, linear slopes from calibration curves, representative images of the pDBS cards calibrated with hemoglobin standards, and effect of sample input volume on the quantitation of hemoglobin, and tables of comparison of four different pDBS card designs, comparison of inter- and intracard variations of pDBS card and unpatterned TFN, percent coefficient of variation, and solvent gradient for HPLC analysis (PDF)
