# Supplementary material for: Patterned Dried Blood Spot Cards for the Improved Sampling of Whole Blood
Source: ACS Meas Sci Au. 2021 Sep 17;2(1):31–8. doi: 10.1021/acsmeasuresciau.1c00031 (PMC8855418; doi:10.1021/acsmeasuresciau.1c00031)
Supplement: Supplementary file 1 — tg1c00031_si_001.pdf [file tg1c00031_si_001.pdf]

Supporting Information

Patterned Dried Blood Spot Cards for Improved Sampling of Whole Blood

Keith R. Baillargeon, Jessica C. Brooks, Philip R. Miljanic and Charles R. Mace*

Department of Chemistry and Laboratory for Living Devices, Tufts University, Medford, MA 02155 USA

* corresponding author email: charles.mace@tufts.edu

Pages: 16

Figures: 5

Tables: 4

Contents: Materials and Methods. Scanned images of card patterning. Schematics of device designs. Tables and graphs depicting device performance, estimation of sample output, calibration curves for the quantitation of amino acids by HPLC-FLD, intercard precision, and complete method details for HPLC analysis.

Materials and Methods

Chemical Reagents and Materials

We purchased Drabkin's reagent, Brij 35 (30% w/w), sodium ICP standard (1000 ppm in 3% nitric acid), sulfosalisylic acid (20% w/v), and ACS reagent grade (ASTM Type I) water from Ricca Chemical. We purchased HPLC grade triethylamine, methanol, acetonitrile, and water from Fisher Scientific. We purchased sodium acetate trihydrate (ACS crystalline), concentrated nitric acid (70% v/v), L-leucine (>98.5%), and O-phosphoric acid (85% ACS certified) from Fisher Scientific. We purchased L(-)-tryptophan (99%) from Acros Organics. We purchased proline and L-(+)-lysine (>98.5%) from Spectrum Chemical. We purchased lyophilized hemoglobin standard from Pointe Scientific (Canton, MI). We purchased the AccQTag ultra derivatization kit from Waters Corporation. We obtained samples of whole blood collected in potassium EDTA vacutainers from Research Blood Components (Watertown, MA). We purchased Munktell TFN, Ahlstrom 226, and Whatman CF-12 grades cellulose papers from Laboratory Sales & Service LLC (Somerville, NJ). We purchased Critoseal vinyl plastic putty from VWR. We purchased 40-mm microhematocrit capillary tubes from LW Scientific. We purchased Fellowes and Avery laminates from Amazon. We purchased ¼" clear acrylic sheets from McMaster Carr. We purchased 2 mL sample vials, bonded red screw caps with silicon septa, and a C18 Zorbax Eclipse Plus column (2.1 x 150 mm, 5 µm) from Agilent Technologies.

Live Subject Statement

We obtained samples of whole blood from Research Blood Components (Watertown, MA). The vendor follows American Association of Blood Banks guidelines for all donors, which includes IRB approved consent to the use of collected blood for research purposes. All research was approved by the Tufts University Institutional Biosafety Committee.

Evaluation of pDBS Card Sampling Accuracy by Quantitation of Hemoglobin

We evaluated the accuracy of our pDBS cards by quantitation of hemoglobin using Drabkin's reagent and a standard protocol.[1] Briefly, we used a standard office hole punch to remove a 6-mm diameter punch from the end of each lateral channel containing whole blood (N=4 replicate punches per card). Then, we submerged each punch in 1.0 mL Drabkin's reagent for 30 minutes before quantifying the concentration of hemoglobin in a 96-microwell plate with a Varioskan LUX microplate reader. Reference values of hemoglobin in samples of whole blood were determined by adding 4 µL of whole blood to 1.0 mL of Drabkin's reagent and incubating for 30 minutes at room temperature. We used lyophilized hemoglobin standards rehydrated with diH_2O (18 MΩ) to construct calibration curves over a range of 3–18 g/dL. We defined the limit of detection (LOD) as the lowest average analyte concentration that could be distinguished from the blank plus two standard deviations.[2] Our blank sample was the Drabkin's assay solution without hemoglobin present (N=20) and our low concentration sample was pure plasma obtained via centrifugation from three different donors (N=20).

We used the methods described above to evaluate the effects of under- and over-filling the pDBS card with the following change. Sample input volume was varied from 60–90 µL in 5 µL increments to simulate under- and over-filling.

Measurement and Adjustment of the Hematocrit

We measured the initial hematocrit of the whole blood sample upon arrival. We added 3 µL of whole blood to a 40-mm microhematocrit capillary tube and sealed the tube at one end with Critoseal putty. We centrifuged the microhematocrit capillary tubes at 1,200 RPM for 3 minutes using a ZipCombo centrifuge from LW Scientific. We obtained images of the microhematocrit tubes using an 8-bit EPSON Perfection V600 PHOTO scanner with a resolution of 800 dpi. We calculated the hematocrit of the sample by measuring the ratio of the length that RBCs occupied

in the tube to the total sample length with ImageJ software.[3] We followed the same procedure for each sample for the measurement of hematocrit (N=2)

We created samples of whole blood at different hematocrit values (20–60%) by adjusting the volume of native plasma in the sample. We confirmed the hematocrit value by measuring the hematocrit value as described above (N=2).

Nitric Acid Digestion of pDBS Samples for the Quantitation of Sodium by ICP-AES

We applied 75 µL of venous whole blood to pDBS cards and allowed them to dry overnight. We pooled four punches (6-mm diameter) from each pDBS card containing approximately 41.2 µL of dried whole blood. Therefore, we prepared liquid reference samples containing 41.2 µL whole blood. We added both samples (pDBS punches and liquid reference) to 5 mL round bottom flasks and digested the samples in 3 mL nitric acid (70% v/v) at 100 °C for 90 minutes. We evaporated the remaining nitric acid from the round bottom flasks. Once cooled, we reconstituted the samples in 3 mL nitric acid (10% v/v) and transferred the contents to 5 mL Eppendorf tubes. We prepared blank samples with 4 punches (6-mm diameter) of unpatterned TFN and followed the digestion procedure above. We calibrated the ICP-AES with liquid calibrants (0.3–10 ppm) before each use. We analyzed all samples using a single phase, high dispersion Prodigy Spec ICP-AES by Leeman Labs Inc. (Hudson, NH).

Quantitation of Amino Acids by HPLC-FLD

We adapted our protocol for the quantitation of amino acids from a published method.[4] Briefly, we deproteinated liquid reference samples of whole blood with 10% sulfosalicylic acid (1:1 v/v), centrifuged (16,000*g*, 3 minutes), and removed the supernatant (20 µL). We adjusted the pH of the liquid reference sample with 60 µL borate buffer (pH 9.7). We removed four punches (6-mm diameter) from pDBS cards. We extracted the dried blood samples in 300 µL methanol (100%) under full-wave sonication using a power sweep ultrasonic cleaner (Shenzhen DeKang

Electronic Cleaning Appliances Co., LTD, China) for 1 hour. Next, we centrifuged the samples (16,000*g*, 3 minutes) and removed the supernatant (paper punch remained in the bottom of the tube). Finally, we dried the sample under a stream of nitrogen and reconstituted it in acetonitrile (50% v/v in water). We derivatized both sample types (liquid reference and dried blood) with 20 µL of the AccQTag reagent and incubated for 10 minutes at 55 °C. Mobile phase A comprised 140 mM sodium acetate and 17 mM triethylamine (pH 5.05). Mobile phase B comprised acetonitrile in water (60% v/v). We analyzed all samples using an Agilent Technologies 1100 series HPLC system accompanied by an Agilent 1100 series fluorescence detector unit (G1321A) and Eclipse Plus C18 column (5 µm, 2.1 x 150 mm). The solvent gradient is summarized in **Table S4**. We used the following methods for each sample: sample injection volume (10 µL), flow rate (0.6 mL/min), column temperature (40 °C), excitation (250 nm) and emission (395 nm). Calibration curves were constructed for each amino acid and fit by linear regression (**Figure S5**).

Figure S1. Patterning thick chromatography papers to control sample distribution and storage. (A) Direct deposition of wax onto thick chromatography papers does not provide complete hydrophobic barriers resulting in uncontrolled sample spreading. (B) Patterning the same papers with a double-sided transfer method forms complete barriers through the thickness of the material and allows unique patterning on each side for enhanced performance. These barriers control the distribution of sample to the four collection zones for storage and future analysis.

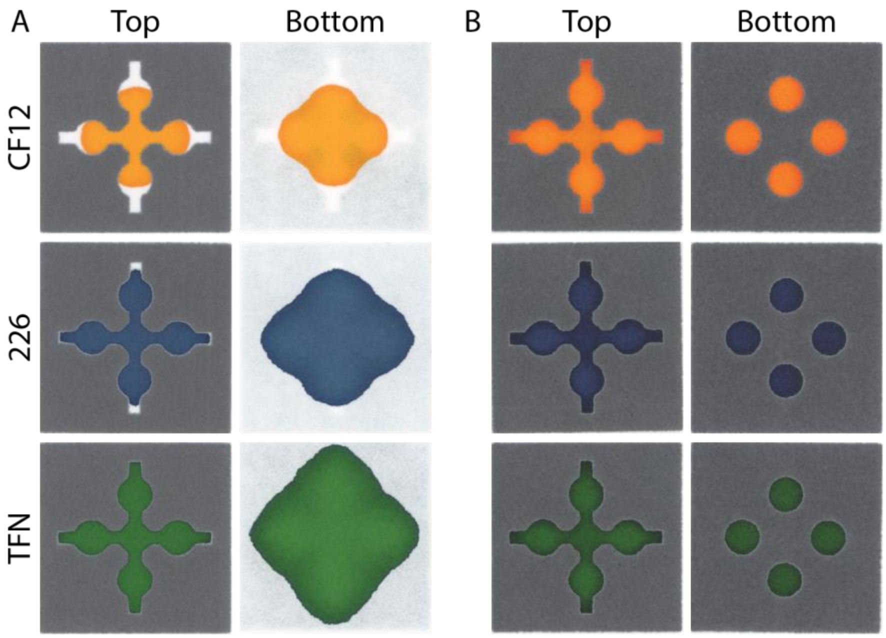

Figure S2. Schematics of various pDBS card designs and assembly strategies. (A) Each device has unique top and bottom patterns to control the distribution of sample. (B) Exploded schematics illustrate the use of transparent laminate sheets to achieve (i) partially sealed, (ii) fully sealed, or (iii) unsealed devices. Laminate was removed prior to punching and eluting from pDBS cards.

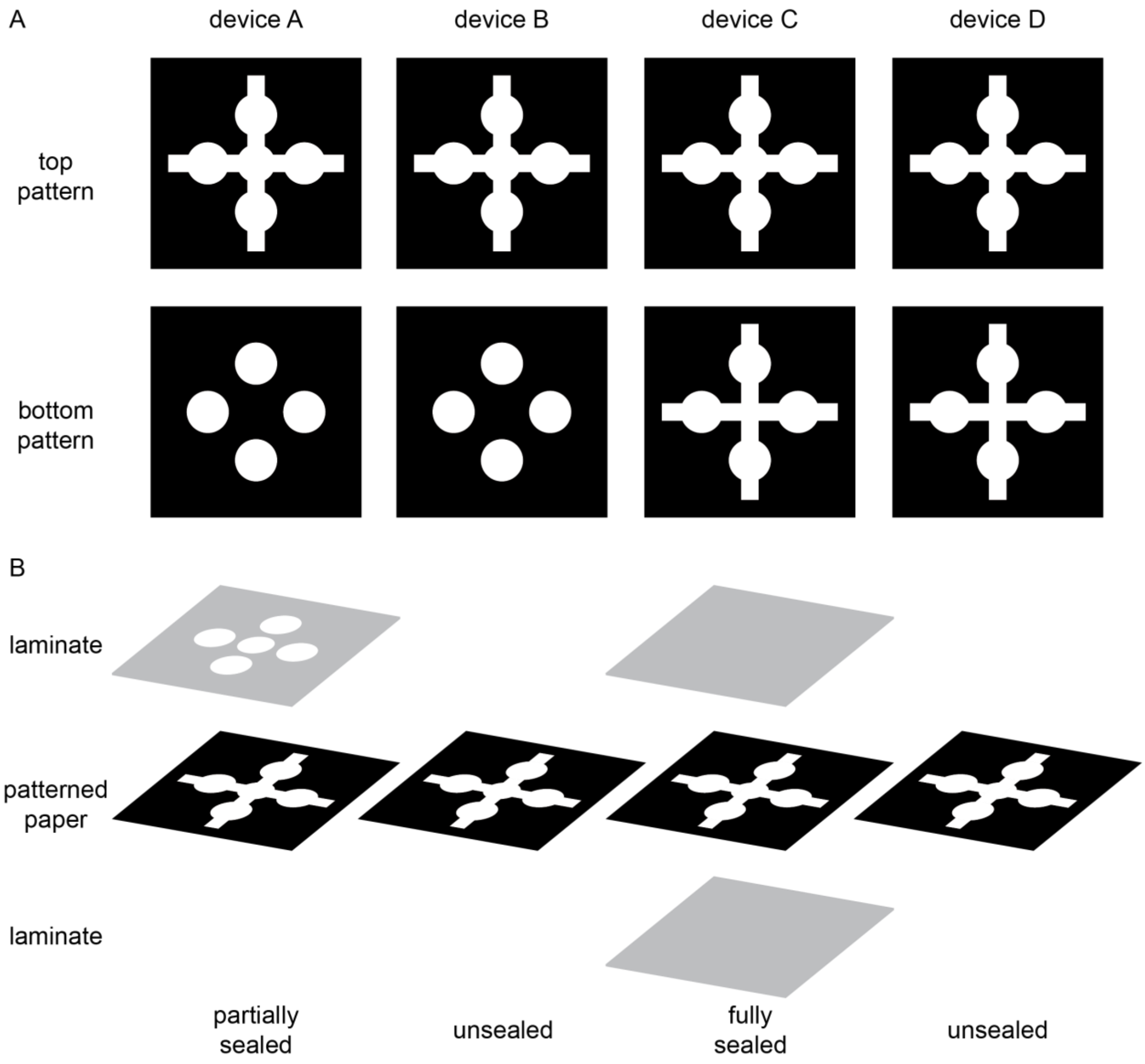

Table S1. Comparison of four different pDBS card designs for the quantitation of hemoglobin at 30% and 50% Hct. Data represent the average of 12 replicates ± standard deviation. Reference hemoglobin values for card design A (30% Hct, 9.6 ± 0.3 g/dL; 50% Hct, 16.0 ± 0.5 g/dL). Reference hemoglobin values for card designs B, C, D (30% Hct, 11.5 ± 0.4 g/dL; 50% Hct, 17.5 ± 0.5 g/dL).

Card Design	30% Hct		50% Hct	
	[Hgb] ± SD (g/dL)	Error	[Hgb] ± SD (g/dL)	Error
A	11.3 ± 0.2	18%	19.8 ± 0.5	24%
B	10.4 ± 0.2	-9%	17.4 ± 0.4	-1%
C	10.2 ± 0.3	-11%	18.4 ± 0.3	5%
D	8.6 ± 0.3	-25%	15.7 ± 0.3	-10%

Figure S3. Estimation of sample volume in pDBS cards. (A) Quantitation of hemoglobin using the Drabkin's assay. The input sample volume of hemoglobin was varied (3–11 µL) over the physiological range (3–18 g/dL hemoglobin). Calibration curves were constructed for each input sample volume (N=1). (B) The linear slope from each calibration curve was plotted as a function of sample volume. (C) pDBS cards were calibrated with hemoglobin standards. The slope of the calibration curve was used to estimate the sample volume. Each data point represents the average of four replicates and error bars represent the standard error of the mean (SEM). (D) Representative images of the pDBS cards calibrated with hemoglobin standards prior to elution.

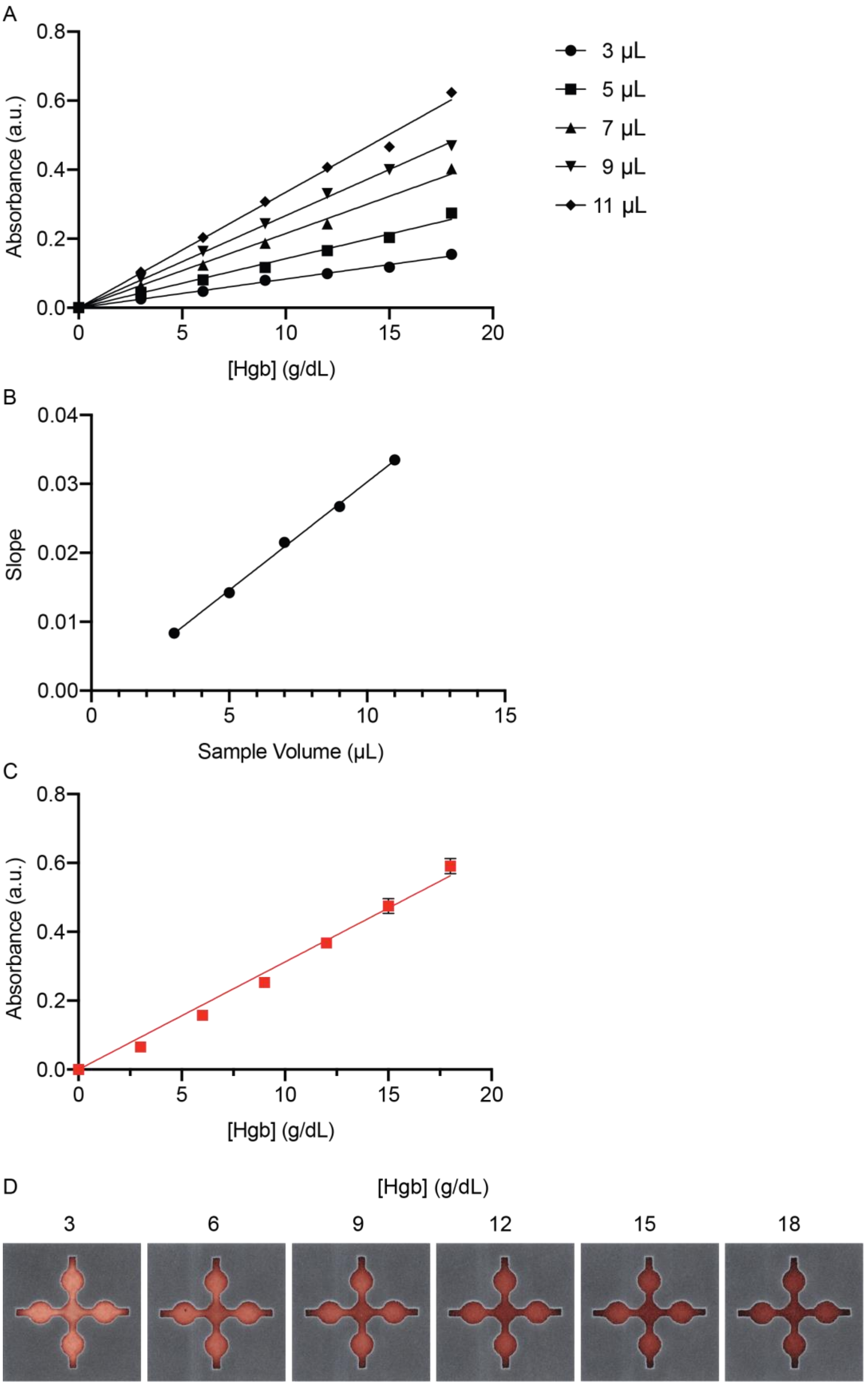
A
Absorbance (a.u.)
0.0
0.2
0.4
0.6
0.8
0
5
10
15
20
[Hgb] (g/dL)
3 μL
5 μL
7 μL
9 μL
11 μL
B
Slope
0.00
0.01
0.02
0.03
0.04
0
5
10
15
Sample Volume (μL)
C
Absorbance (a.u.)
0.0
0.2
0.4
0.6
0.8
0
5
10
15
20
[Hgb] (g/dL)
D
[Hgb] (g/dL)
3
6
9
12
15
18

Figure S4. Effect of sample input volume on the quantitation of hemoglobin in pDBS cards. (A) Each data point represents the average of three replicates and error bars represent the standard error of the mean. The liquid blood reference sample had a hemoglobin concentration of 18.1 ± 0.4 g/dL (dashed line). (B) Representative images of pDBS cards under- (< 75 µL) and over-filled (> 75 µL).

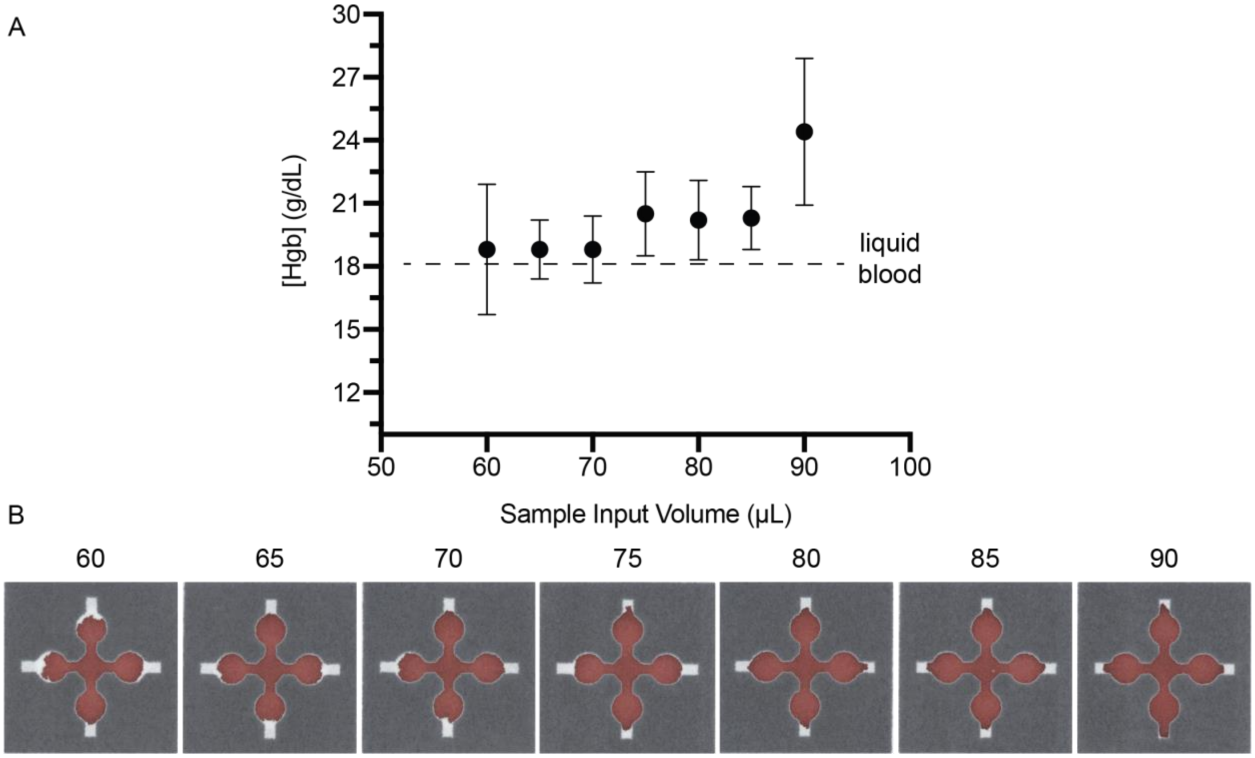

Table S2. Comparison of inter- and intra-card variation of pDBS card and unpatterned TFN at various hematocrit values. Data represent the average of 20 replicates ± standard deviation (SD).

	pDBS Card			Unpatterned TFN		
Hct	[Hgb] (g/dL)	Inter-card SD	Intra-card SD	[Hgb] (g/dL)	Inter-card SD	Intra-card SD
20%	6.2	4%	4%	5.3	5%	5%
30%	9.4	4%	4%	9.1	6%	5%
40%	12.3	4%	4%	12.3	5%	5%
50%	14.7	5%	4%	15.2	5%	5%
60%	18.2	4%	4%	17.4	4%	5%

Figure S5. Calibration curves were constructed using HPLC grade amino acid standards (N=1). All data were fit using linear regression in Prism Graphpad. (A) Leucine (slope 3293.9; R^2 0.9993). (B) Proline (slope 794.5; R^2 0.9997). (C) Lysine (slope 1319.5; R^2 0.9987). (D) Tryptophan (slope 2559.6; R^2 0.9987).

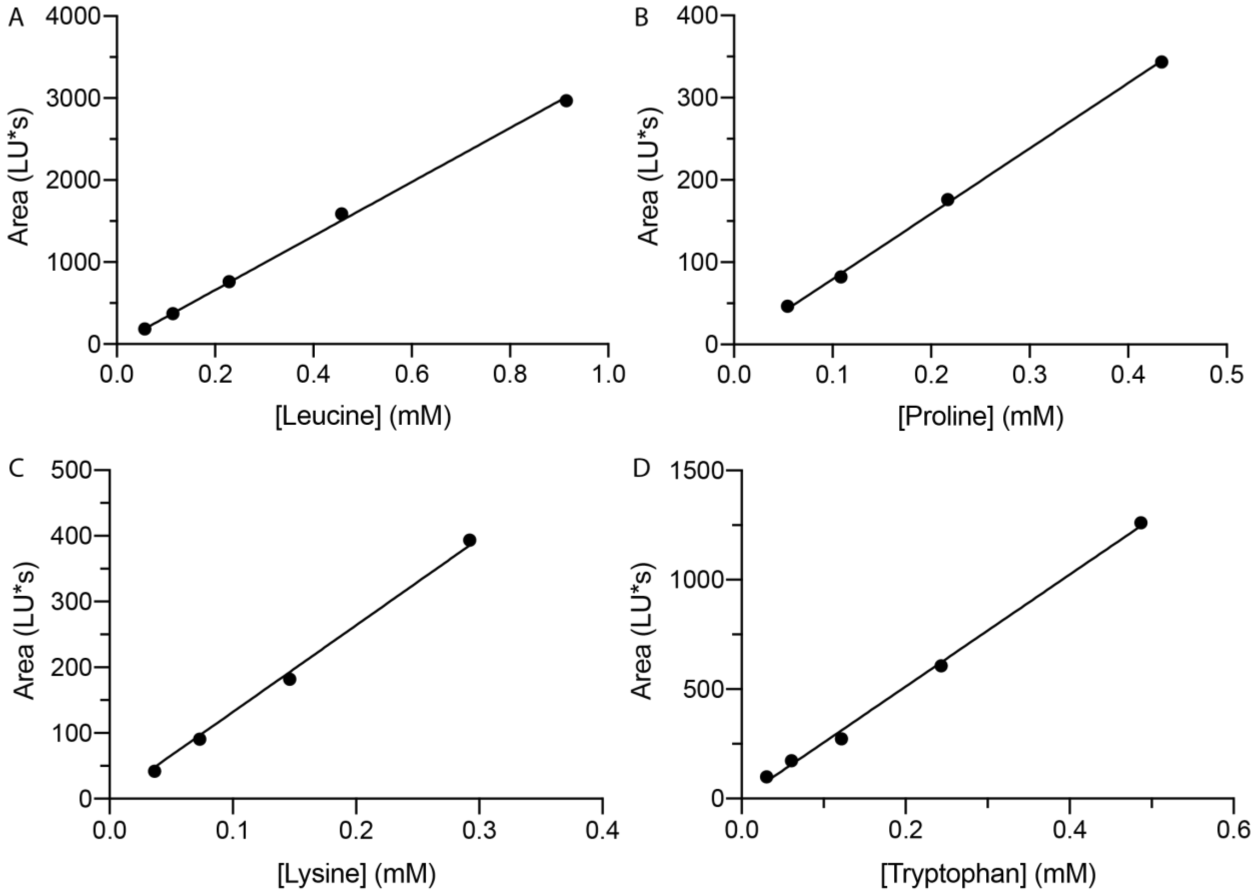

Table S3. Percent coefficient of variation (%CV) for pDBS and reference samples at 20% and 40% hematocrit. %CV was calculated as the ratio of standard error of the mean (SEM) to average value for each amino acid.

	20% Hematocrit		40% Hematocrit	
	pDBS	Reference	pDBS	Reference
[Trp]	5.2%	4.0%	0.8%	3.6%
[Leu]	6.7%	5.6%	2.6%	2.8%
[Pro]	5.5%	6.7%	1.0%	3.5%
[Lys]	5.4%	5.6%	5.7%	5.0%

Table S4. Solvent gradient for HPLC analysis.

Time (min)	solvent A (%)	solvent B (%)
0	100	0
0.5	98	2
15	93	7
19	87	13
33	68	32
34	0	100
39	0	100
40	100	0
50	100	0

References

1. Baillargeon, K. R.; Murray, L. P.; Deraney, R. N.; Mace, C. R. High-yielding separation and collection of plasma from whole blood using passive filtration. *Anal. Chem*., **2020**, *92*, 16245–16252.
2. Armbruster, D. A.; Pry, T. Limit of blank, limit of detection and limit of quantitation. *Clin. Biochem. Rev.,* **2008**, *29*, S49–S52.
3. Schneider, C. A.; Rasband, W. S.; Eliceiri, K. W. NIH image to imageJ: 25 years of image analysis. *Nat. Methods,* **2012**, *9*, 671–675.
4. Bloom, K.; Meyers, G. D.; Bennett, M. J. A quantitative method for the measurement of dried blood spot amino acids using ultra-performance liquid chromatrography. *J. Appl. Lab. Med.,* **2016**, *1*, 271–279.
